# The Voltage-Gated Potassium Channel *Shal* (K_v_4) Contributes to Active Hearing in *Drosophila*

**DOI:** 10.1523/ENEURO.0083-24.2024

**Published:** 2025-01-03

**Authors:** Eli S. Gregory, YiFeng Y. J. Xu, Tai-Ting Lee, Mei-ling A. Joiner, Azusa Kamikouchi, Matthew P. Su, Daniel F. Eberl

**Affiliations:** ^1^Department of Biology, University of Iowa, Iowa City, Iowa 52242; ^2^Graduate School of Science, Nagoya University, Nagoya 464-8602, Japan; ^3^Institute of Transformative Bio-Molecules, Nagoya University, Nagoya 464-8601, Japan; ^4^Institute for Advanced Research, Nagoya University, Nagoya 464-8601, Japan

**Keywords:** active hearing mechanism, Kv4, mechanotransduction, sensory cilia, Shal, voltage-gated potassium channel

## Abstract

The full complement of ion channels which influence insect auditory mechanotransduction and the mechanisms by which their influence is exerted remain unclear. *Shal* (K_v_4), a *Shaker* family member encoding voltage-gated potassium channels in *Drosophila melanogaster*, has been shown to localize to dendrites in some neuron types, suggesting the potential role of *Shal* in *Drosophila* hearing, including mechanotransduction. A GFP trap was used to visualize the localization of the *Shal* channel in Johnston's organ neurons responsible for hearing in the antenna. *Shal* protein was localized strongly to the cell body and inner dendritic segment of sensory neurons. It was also detectable in the sensory cilium, suggesting its involvement not only in general auditory function but specifically in mechanotransduction. Electrophysiological recordings to assess neural responses to auditory stimuli in mutant *Shal* flies revealed significant decreases in auditory responses. Laser Doppler vibrometer recordings indicated abnormal antennal free fluctuation frequencies in mutant lines, indicating an effect on active antennal tuning, and thus active transduction mechanisms. This suggests that *Shal* participates in coordinating energy-dependent antennal movements in *Drosophila* that are essential for tuning the antenna to courtship song frequencies.

## Significance Statement

The study of fruit fly hearing has revealed mechanosensitive ion channels that participate in mechanotransduction, and as in mammalian hearing, energy-dependent mechanisms actively amplify and tune auditory processes. Identifying distinct roles played by different ion channels is essential to better understand this process. Here, we explore the influence of a specific voltage-gated potassium channel, *Shal*, on fly hearing and find that it affects specific parts of the mechanotransduction process. Our research uncovers *Shal*'s localization in sensory dendrite regions of auditory neurons, where it contributes to shaping mechanotransduction and active antennal tuning. Understanding *Shal*'s involvement in auditory function and mechanotransduction deepens our knowledge of fly hearing and unveils a key player in the coordination of energy-dependent active antennal movements.

## Introduction

Several ion channels participating in insect auditory mechanosensory transduction have been identified, but the precise transduction mechanisms are still poorly understood. For example, the TRPN channel, encoded by the *no mechanotransduction potential C* (*NompC*; Walker et al., 2000; Göpfert and Robert, 2003; Effertz et al., 2011) gene in *Drosophila*, and the TRPV channel, comprising two subunits encoded by *inactive* (*iav*) and *nanchung* (*nan*) genes (Kim et al., 2003; Gong et al., 2004; B. Li et al., 2021), are central to mechanotransduction, but their precise contributions are yet to be fully established. These channels localize to the sensory cilia of chordotonal organs, also called scolopidia, which make up Johnston's organ (JO) in the antenna, with NompC localizing distal to the ciliary dilation (Lee et al., 2010) and Iav/Nan localizing in the ciliary segment proximal to the ciliary dilation (Kim et al., 2003; Gong et al., 2004). Different lines of evidence support two prevailing models of auditory mechanotransduction, known as the NompC model and the Iav/Nan model (Eberl et al., 2016; Hehlert et al., 2021). In the NompC model, TRPN and TRPV act in series, with the NompC channel functioning as the primary mechanotransduction channel to provide the initial transduction current and Iav/Nan required for propagation as well as providing additional mechanosensitivity. In contrast, the Iav/Nan model attributes mechanosensitivity to Iav/Nan, with NompC acting in parallel to control amplification gain (Albert and Göpfert, 2015; Eberl et al., 2016; Hehlert et al., 2021).

The sensory dendrite, in addition to being the site of the initial transduction event to generate receptor potentials in response to mechanosensory stimulation, is also involved in active mechanisms. By inserting energy into spontaneous movements of the antenna (Göpfert and Robert, 2003; Göpfert et al., 2005), sensory responses to low amplitude stimulation can be amplified, and antennal movements are tuned to enhance reception of sound frequencies related to the *Drosophila* courtship song (Riabinina et al., 2011). These active movements are thought to arise from two sources: first, energy representing reduced mechanical compliance upon transduction channel closing, returning kinetic energy into movement of the antenna (Albert et al., 2007; Nadrowski et al., 2008; Effertz et al., 2012), second, active ciliary movement generated by the ATP-dependent axonemal dynein motors in the proximal segment of the sensory cilium (Eberl, 1999; Karak et al., 2015). It is unclear if and how additional ion channels beyond NompC/Nan/Iav may contribute to the excitability of the sensory cilia in JO, though a number of promising targets exist. This includes the voltage-gated potassium channel encoded by the *Shal* gene, orthologous to K_v_4 (Butler et al., 1990; Tsunoda and Salkoff, 1995; Ping et al., 2011), previously shown in other neurons to localize to dendrites rather than axons (Diao et al., 2010) and expressed in JO neurons of adult flies (Fly Cell Atlas; H. Li et al., 2022). If Shal indeed localizes in the dendrites, it has the potential to contribute to shaping JO neuron receptor potentials.

Here we first demonstrate that *Shal* is expressed in JO neurons and localizes to the sensory dendrites, including the sensory cilium. Moreover, we show that *Shal* loss-of-function genotypes result in severe reductions in auditory function, as determined by electrophysiology from the antennal nerve. Furthermore, we found using laser Doppler vibrometry (LDV) that these genotypes disrupt the active mechanisms in JO neurons, especially impairing the active tuning to courtship song frequencies. The K_v_4 channel encoded by *Shal* thus plays a key role in active transduction mechanisms in insect hearing.

## Materials and Methods

### Fly strains

*Drosophila melanogaster* genotypes used in this study are listed in Table 1. Controls included the wild-type Canton S strain, as well as *TM3*, *Sb* heterozygotes from the *Shal^MI00446^* strain, and, in some experiments, *w elav^C155^;; TM6B, Tb Hu/+* flies resulting from outcrossing the balanced dominant-negative flies listed in the table with a cantonized *w^1118^* strain. In all experiments, the different controls were tested to ensure no significant differences before pooling. For all experiments, *Shal^MI00446^* flies were homozygotes selected from the balanced stock, and dominant-negative Shal flies were of the genotype shown in the table. For each experiment, both male and female flies were used, with sex noted in primary data, though no differences were seen between sexes.

**Table 1.** *Drosophila* genotypes used in this study

Line	Research resource identifier	References
Canton S (Hotta)		
*w^1118^; PBac{WH}Shal^f00495^*	RRID: BDSC_18338	(Thibault et al., 2004)
*w^1118^; Mi{ET1}Shal^MB05249^*	RRID: BDSC_24326	(Metaxakis et al., 2005; Bellen et al., 2011)
y^1^ w*; Mi{MIC}Shal^MI00446^/TM3, Sb	RRID: BDSC_31006	(Venken et al., 2011)
y^1^ w*; Mi{MIC}Shal^MI10881^	RRID: BDSC_56089	(Venken et al., 2011)
y^1^ w*; Mi{PT-GFSTF.1}Shal^MI00446-GFSTF.1^	RRID: BDSC_60149	(Nagarkar-Jaiswal et al., 2015)
*w elav^C155^-Gal4;; UAS-HA-Shal^W362F^/TM6B, Tb Hu*		(Ping et al., 2011)

### Antibody staining and imaging

*Drosophila* pupal heads were dissected in cold phosphate-buffered saline (PBS) and then fixed in 4% paraformaldehyde for 15 min. After three washes in PBS with 0.2% Tween-20 (PBT) with rotation over 30 min, the samples were blocked with blocking buffer (BB; freshwater fish skin gelatin, normal goat serum, and bovine serum albumin in PBT) for 1 h with rotation. Primary antibodies diluted in BB included mouse anti-FLAG antibody (1:100; Sigma-Aldrich; RRID:AB_796202), mouse anti-GFP antibody (1:500; Thermo Fisher Scientific; RRID:AB_2335261), anti-HA rMs-IgG1 (1:500; Developmental Studies Hybridoma Bank; RRID:AB_3105930), and rabbit anti-HRP antibody (1:500; Cappel). Primary antibody incubation took place overnight at 4°C with rotation.

The following day, the samples were washed three times in PBT over 30 min with rotation and then incubated with secondary antibodies and phalloidin for 2 h at room temperature with rotation. Secondary antibody diluted in BB (1:500) consisted of goat anti-mouse Oregon Green (488; Thermo Fisher Scientific; RRID:AB_2539797) and goat anti-rabbit TRITC (Jackson Immunoresearch Laboratories; RRID:AB_261740), along with phalloidin 405 (1:1,000; Thermo Fisher Scientific). Samples underwent two 15 min washes in PBT with rotation followed by a brief 5 min wash in PBS. Finally, samples were mounted in Fluoromount (Thermo Fisher Scientific; RRID:SCR_015961) onto glass slides with 1.5 coverslips and imaged with a Leica STELLARIS 8 confocal microscope, using a 63× objective lens with oil immersion. Images were adjusted by applying brightness and contrast changes uniformly to the entire image in each channel using the Fiji software (RRID: SCR_002285).

### Electrophysiology

Sound-evoked potentials (SEPs) were captured using a pair of electrolytically sharpened tungsten recording electrodes (Eberl et al., 2000; Eberl and Kernan, 2011). The recording electrode was inserted between the first and second segments of the antennae, while the reference electrode was inserted into the head cuticle near the posterior orbital bristle. A computer-generated pulse song was introduced frontally to the fly under near-field conditions.

Signals were subtracted and amplified with a differential amplifier (DAM50, World Precision Instruments) and digitized at 10 kHz (USB-6001, National Instruments). Average response values were measured as the max–min values in an averaged trace from 10 consecutive presentations of the described protocol.

### LDV data collection

Following 2 d of entrainment in a 12:12 LD regime, male and female *Drosophila* aged between 3 and 10 d old were aspirated into micropipette tips as for electrophysiology experiments. Fly head movement was restricted by the application of modeling clay to the edge of the pipette. Blue light-cured glue was then applied to the entirety of the right antennae (to completely inhibit movement) as well as the base of the left antennae.

The tip with the immobilized fly was then attached to a rod held in a micromanipulator on a vibration isolation table in a temperature-controlled room (set to 25 ± 1°C). The fly was positioned such that its left arista was perpendicular to the beam of a laser Doppler vibrometer (VibroFlex, Polytec).

Unstimulated aristal vibrations [denoted as “free fluctuations” (FF)] were first recorded while the fly was awake. The fly was then sedated via continuous CO_2_ exposure for 2 min, before another free fluctuation recording was made. These recordings allowed for investigating both active (awake) and passive (sedated) hearing states.

### LDV data analysis: mechanical tuning calculation

Fast Fourier transforms of recording values were made using the VibSoft Polytec software for frequencies from 1 to 10 kHz. Frequency values below 100 Hz were excluded from analyses due to significant noise in the recordings.

A forced damped oscillator function was applied to transformed data via the lme4 package (version 1.1-33) in R (version 4.3.0) as follows:

$$\dot{X}(\omega )=\;\frac{\omega .\frac{F_{0}}{m}}{\sqrt{\left({\left(\omega_{0}^{2}-\;\omega^{2}\right)}^{2}+{\left(\omega .\frac{\omega_{0}}{Q}\right)}^{2}\right)}},$$

where *F*_0_ is the external force strength, *m* is the flagellar apparent mass, *ω* is the angular frequency, *ω*_0_ is the natural angular frequency, and *Q* is the quality factor, *mω*_0_/*γ* (*γ*, damping constant).

This equation used by Göpfert et al. (2005), here derived for $\dot{X}(\omega )$, allowed for estimation of the natural angular frequency of recordings from both active and passive states for each fly, enabling calculation of the mechanical tuning frequency, *f*_0_ (*ω*_0_/2π).

### LDV data analysis: power gain calculation

Following Göpfert et al. (2005), power gain calculations utilized the results of the forced damped oscillator function fits by enabling the calculation of the ratio of total fluctuation power of an individual's active and passive states.

We defined power gain as follows:

$$\mathrm{Power}\;\mathrm{gain}=\;\frac{\omega_{a}^{2}\langle x_{a}^{2}\rangle }{\omega_{p}^{2}\langle x_{p}^{2}\rangle }-1,$$

where $\omega_{a}$ is the natural angular frequency of active system, $\omega_{p}$ is the natural angular frequency of passive system, $\left\langle x_{a}^{2}\right\rangle$ is the sum of squared Fourier displacement amplitudes in active state, and $\left\langle x_{p}^{2}\right\rangle$ is the sum of squared Fourier displacement amplitudes in passive state.

After fitting the above damped harmonic oscillator function to raw velocity data and extracting fit parameters, these parameters were used to calculate velocity estimates for the curve fit between 1 and 10,000 Hz. These velocity values were then converted to displacement values using the formula *X*(*ω*) = $\dot{X}(\omega )$/*ω* and then squared. These squared displacement values were then used to calculate the sums of squared Fourier displacement amplitudes per individual fly.

Natural angular frequency values were calculated from the function fits, while sums of squared Fourier displacement amplitudes were estimated from the following:

$$\langle x_{i}^{2}\rangle =\int_{0}^{\infty }x_{i}^{2}(\omega )d\omega .$$

### Statistical analysis

To evaluate statistical differences in mutant SEP data compared with controls, we pooled three control genotypes (see above, Fly strains) after testing for statistical differences by ANOVA. Analysis of mutant genotypes compared with controls utilized Brown–Forsythe ANOVA, given that the data were largely Gaussian but showed heterogeneity of variances among groups. Dunnett's T3 multiple-comparison post hoc test was then applied to compare each group with every other group, with adjustments for multiple comparisons to control the overall Type 1 error rate. For LDV data, Arista Best Frequencies for both awake and sedated conditions were analyzed in the same way as the SEP data. However, power gain and *Q* were analyzed using Kruskal–Wallis ANOVA, a nonparametric alternative, because the data were significantly non-Gaussian. This was followed by Dunn's multiple-comparison post hoc test to determine specific differences between each experimental group and the pooled control. All statistical analysis and graphing used GraphPad Prism version 9.5.1 (GraphPad Software). In addition, estimation statistics (https://www.estimationstats.com/) were conducted for all measures comparing mutant genotypes to controls with data summarized in Table 2.

**Table 2.** Estimation statistics

Genotype	*N*	Mean	SD	Mean difference from control	95% confidence intervals of difference
SEPs (Fig. 2*C*)					
Control	56	913.9	126.8		
*Shal^MI00446-GFSTF.1^*	9	974.4	72.7	60.6	13.3–126
*Shal^MI00446^*	9	332.4	45.7	−581	−630 to −543
*Shal^f00495^*	8	241.1	31.8	−673	−715 to −638
*Shal^DN^*	10	299.6	61.3	−614	−668 to −570
*Shal^MI10881^*	8	425.8	36.9	−488	−534 to −453
*Shal^MB05249^*	10	390.2	38.0	−524	−566 to −487
Arista best freq. (awake; Fig. 3*B*)					
Control	25	240.9	45.5		
*Shal^MI00446-GFSTF.1^*	10	273.3	61.5	32.4	2.2–88
*Shal^MI00446^*	17	340.9	36.1	100	74.0–123
*Shal^f00495^*	11	423.7	52.5	183	151–219
*Shal^DN^*	11	338.9	78.2	98.1	57.4–150
*Shal^MI10881^*	10	197.0	78.2	−43.9	−83.5 to 18.4
*Shal^MB05249^*	10	260.6	88.0	19.7	−21.5 to 98.2
Arista best freq. (sedated; Fig. 3*B*)					
Control	25	797.9	94.0		
*Shal^MI00446-GFSTF.1^*	10	827.5	83.6	29.6	−22.2 to 106
*Shal^MI00446^*	17	873.1	68.1	75.2	25.9–122
*Shal^f00495^*	11	758.0	84.2	−39.9	−90.4 to 26.6
*Shal^DN^*	11	737.7	29.0	−60.1	−99.5 to −21.1
*Shal^MI10881^*	10	856.0	155	58.1	−36.9 to 161
*Shal^MB05249^*	10	796.3	80.9	−1.5	−60 to 56.6
Power gain (Fig. 3*C*)					
Control	25	7.32	7.9		
*Shal^MI00446-GFSTF.1^*	10	13.6	20.3	6.3	−1.7 to 28
*Shal^MI00446^*	17	20.1	25.0	12.8	3.7–30
*Shal^f00495^*	11	2.61	2.8	−4.7	−8.6 to −1.6
*Shal^DN^*	11	2.82	2.1	−4.9	−8.5 to −1.6
*Shal^MI10881^*	10	6.43	4.8	−0.9	−5.2 to 3.1
*Shal^MB05249^*	10	2.60	4.1	−4.7	−8.4 to −0.7
*Q* (awake; Extended Data Fig. 3-1*A*)					
Control	25	1.20	0.43		
*Shal^MI00446-GFSTF.1^*	10	1.80	0.46	0.60	0.31–0.96
*Shal^MI00446^*	17	2.66	1.74	1.46	0.83–2.54
*Shal^f00495^*	11	0.68	0.17	−0.52	−0.75 to −0.36
*Shal^DN^*	11	0.43	0.13	−0.77	−1.00 to −0.62
*Shal^MI10881^*	10	1.47	2.02	0.28	−0.45 to 2.39
*Shal^MB05249^*	10	1.26	0.64	0.06	−0.25 to 0.62
*Q* (sedated; Extended Data Fig. 3-1*B*)					
Control	25	0.96	0.20		
*Shal^MI00446-GFSTF.1^*	10	0.94	0.23	−0.02	−0.15 to 0.15
*Shal^MI00446^*	17	1.02	0.26	0.06	−0.07 to 0.19
*Shal^f00495^*	11	1.00	0.17	0.04	−0.08 to 0.14
*Shal^DN^*	11	1.01	0.18	0.05	−0.11 to 0.15
*Shal^MI10881^*	10	1.04	0.21	0.08	−0.05 to 0.23
*Shal^MB05249^*	10	1.11	0.20	0.15	−0.01 to 0.26

## Results

### Shal is expressed in JO neurons and localizes to somata and dendrites

In *Drosophila* single-cell RNAseq data, *Shal* is expressed broadly in JO neurons at the adult stage (H. Li et al., 2022; Extended Data Fig. 1-1). A tagged form of *Shal*, when expressed in olfactory projection neurons, has been shown to localize to the soma and dendrites, but not to axons (Diao et al., 2010). Furthermore, K_v_4.2 in the rat hippocampus has also been found to localize to dendrites (Sheng et al., 1992). Thus, we tested whether Shal channels in JO neurons are localized in the sensory dendrites, where they could contribute to active mechanosensation.

**Figure 1.**
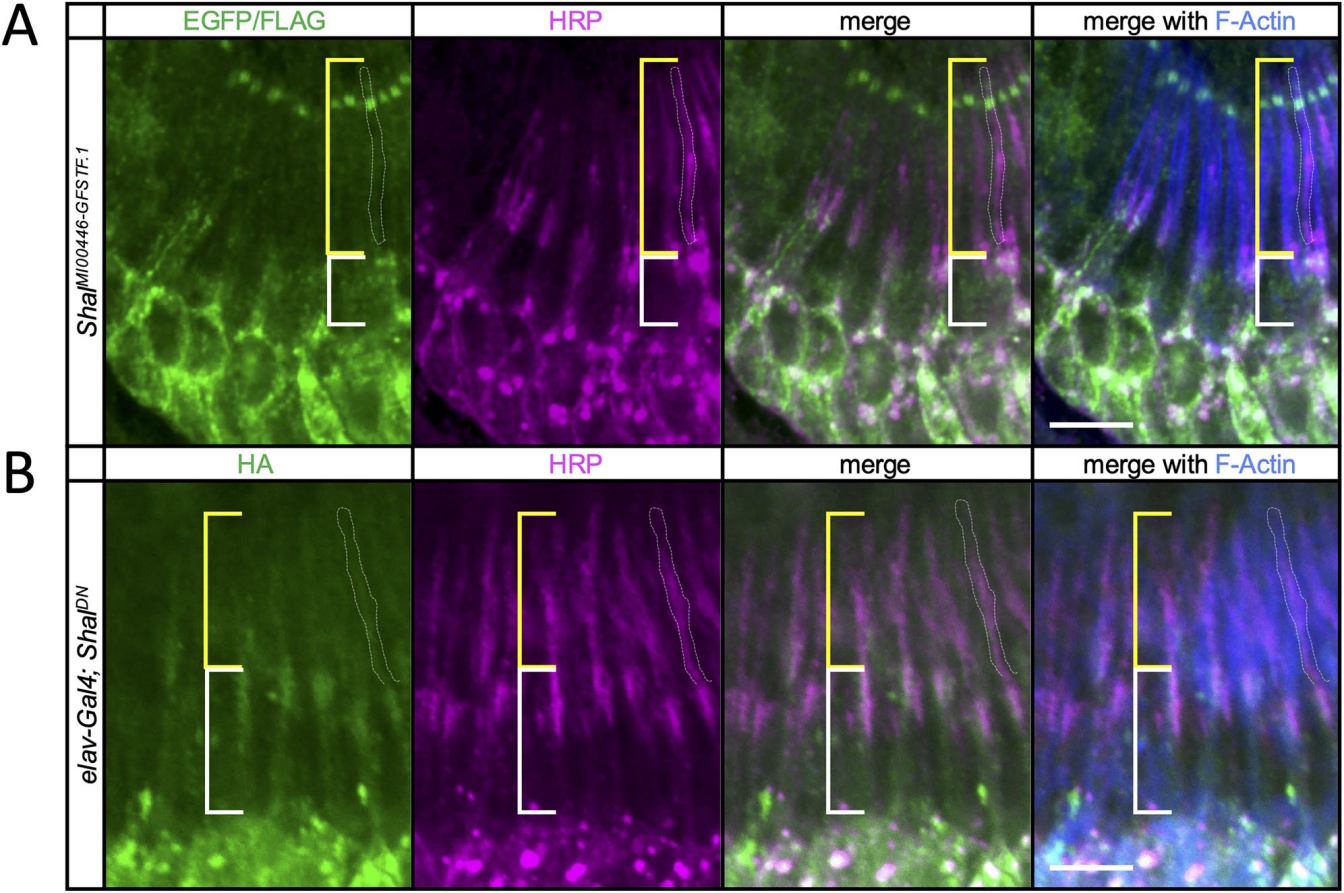
Shal is expressed in JO neurons and localizes to sensory dendrites and somata. ***A***, Immunostaining of pupal JO from the Shal protein trap line, *Shal^MI00446-GFSTF.1^*. The tagged Shal protein is stained with anti-EGFP and anti-FLAG in the green channel. Neurons are visualized with anti-HRP (magenta) which shows enhanced dendrite staining. Phalloidin (blue) stains the scolopale rods in the scolopale cell surrounding the sensory dendrite. Brackets indicate the inner (white bracket) and outer (yellow bracket) dendritic segments. White-dotted outline indicates an example cilium. Scale bar, 5 μm. ***B***, Immunostaining of pupal JO from flies expressing the Shal dominant-negative construct, *UAS-HA-Shal^W362F^*, in neurons. Anti-HA staining shows the dominant-negative construct in green, with anti-HRP (magenta) and phalloidin (blue). Brackets and white-dotted outline as in ***A***. Scale bar, 5 μm. For expression of Shal in the Fly Cell Atlas antennal single nucleus RNA sequencing data, see Extended Data Figure 1-1.

We first used a Shal protein trap line, *Shal^MI00446-GFSTF.1^*, in which GFP and other tags are fused in-frame with the Shal coding sequence in the endogenous locus (Nagarkar-Jaiswal et al., 2015). We found staining for this tagged Shal protein in JO neurons. While staining is strongest in the membrane of JO neuron somata, Shal staining was also detected at lower intensity in the cilium (Fig. 1*A*), as indicated relative to the anti-HRP marker which labels neurons, especially the dendrites.

10.1523/ENEURO.0083-24.2024.f1-1 Figure 1-1 **Expression of *Shal* in antennal single nucleus RNA sequencing (Fly Cell Atlas)** A. Annotated clustering of single-nucleus RNA transcript expression from antenna (reproduced from Li et al. (2022) with permission), showing a cluster of cells representing the JO neurons (circled). B. Expression of *Shal* (red) depicted over the same clusters indicates that *Shal* is expressed in JO neurons (circled) as well as olfactory neurons, using SCope (https://scope.aertslab.org/) (Davie et al., 2018). Download Figure 1-1, TIF file.

To confirm this localization, we also used an HA-tagged dominant–negative construct (Table 1; Ping et al., 2011) expressed in all neurons with the *elav^C155^-Gal4* driver. Anti-HA also showed staining in this genotype in the dendrites colinear with the anti-HRP marker (Fig. 1*B*). Thus, the expression pattern of Shal suggests a physiological role in JO neurons, and its localization to the sensory cilium is consistent with a possible role in sensory transduction mechanisms.

### Shal is required for proper auditory function in *Drosophila*

**Figure 2.**
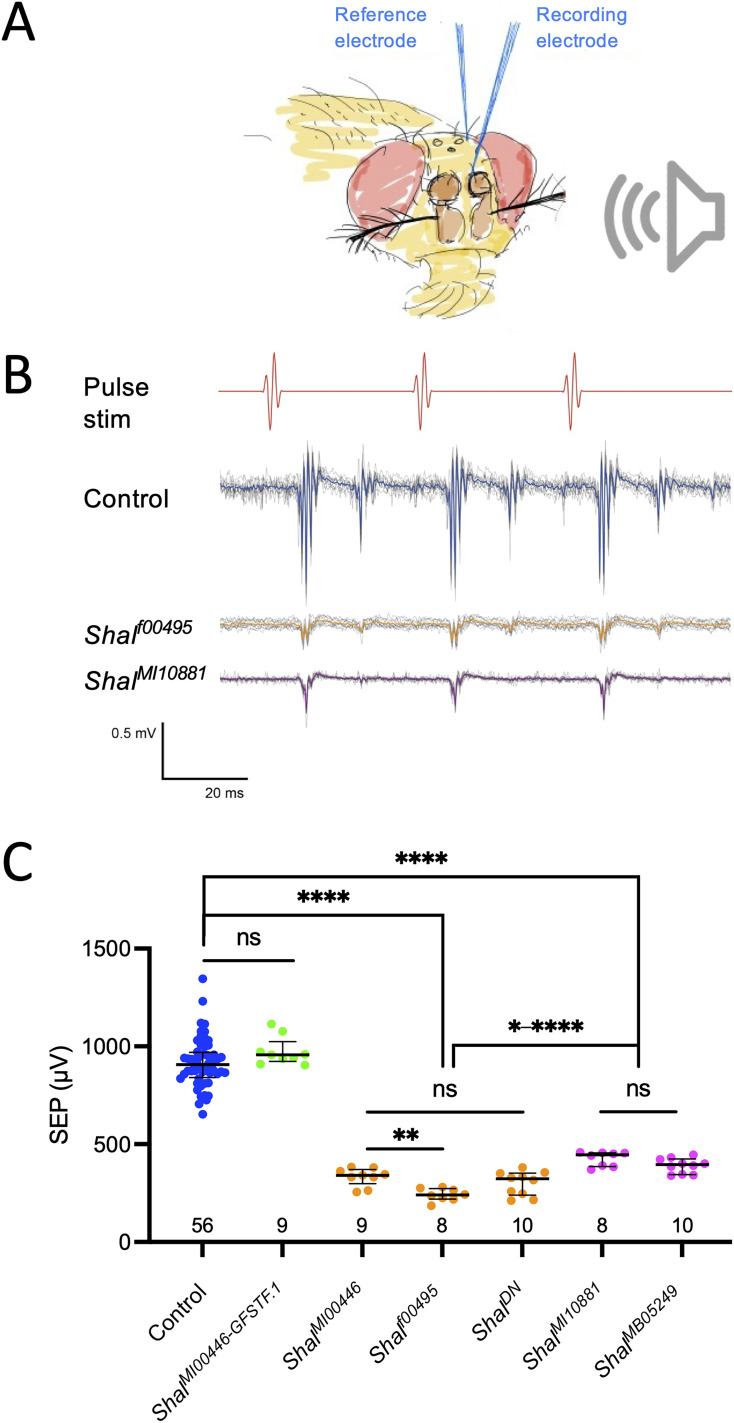
*Shal* loss of function impairs auditory signals in the antennal nerve. ***A***, Schematic of electrophysiological recording preparation. SEPs are recorded as differential field potentials from a tungsten electrode placed near the antennal nerve (recording electrode) relative to one inserted in the dorsal head capsule (reference electrode) in response to presentation of near-field acoustic stimuli. ***B***, Example traces from control and mutant flies in response to synthetic pulse song stimulus. Individual responses to 10 consecutive stimuli are depicted as thin gray lines and their average as the thicker blue (control), orange (mutant), or magenta (mutant) line, in agreement with the color scheme in ***C***. ***C***, Scatterplot of SEP amplitudes recorded from flies with *Shal*-related genotypes. Smaller delayed responses represent acoustic echo artifacts. Each dot represents the SEP amplitude recorded from one antenna, and the number of antennae tested for each genotype is indicated at the bottom of the graph. Bars indicate means, and error bars represent SEM. Controls (blue dots) and the Shal protein trap flies, *Shal^MI00446-GFSTF.1^* (green dots), are not significantly different, but all other genotypes are significantly different from controls. Strong alleles (orange dots) produce significantly lower SEPs than weak alleles (magenta dots). The dominant-negative *Shal* genotype (*Shal^DN ^*= *w elav^C155^-Gal4;; UAS-HA-Shal^W362F^/TM6B, Tb Hu*) behaves as a strong mutant genotype. Brown–Forsythe ANOVA; *p* < 0.0001; with Dunnett's multiple comparisons (ns, not significant; *p < 0.05; **p < 0.01; ***p < 0.001; ****p < 0.0001). For the map of the Shal locus and insertional constructs of Shal alleles, see Extended Data Figure 2-1.

To test whether auditory function was impaired following *Shal* mutation, we tested several *Shal* loss-of-function genotypes (Table 1, Extended Data Fig. 2-1) for changes in SEPs (Eberl et al., 2000; Eberl and Kernan, 2011). In this electrophysiological assay, field potentials recorded from the antennal nerve at the joint between segments 1 and 2 represent the combined auditory signals in the axons from all JO neurons (Fig. 2*A*).

10.1523/ENEURO.0083-24.2024.f2-1 Figure 2-1 **Map of *Shal* locus.** Upper panel shows a screenshot of the JBrowse genome browser depicting the *Shal* locus on chromosome 3L. *Shal* is transcribed in the leftward direction, with three transcript splice isoforms (coding regions in orange boxes, non-coding regions in gray). Transposon insertion sites are depicted by small blue triangles, labeled. Corresponding transposon structures are diagrammed below (from the Gene Disruption Project (https://flypush.research.bcm.edu/pscreen/transposons.html)), with orientation information relative to the map. Download Figure 2-1, TIF file.

In contrast to controls, *Shal* insertion mutants all showed significantly reduced SEP amplitudes (Fig. 2*B*,*C*; Brown–Forsythe ANOVA; *p* < 0.0001; Table 2). Compared with controls, two insertion alleles, *Shal^MI00446^* and *Shal^f00495^*, showed the most reduced SEPs (Fig. 2*C*; Dunnett's T3 multiple comparisons; *p* < 0.0001 each; Extended Data Fig. 2-1; Table 2), while *Shal^MI10881^* and *Shal^MB05249^* showed SEPs that were significantly reduced compared with controls (Dunnett's; *p* < 0.0001 each; Table 2) but significantly higher than the other two alleles (Fig. 2*B*,*C*; Dunnett's; *p* values between 0.006 and <0.0001; Extended Data Fig. 2-1). To test for possible changes in hearing function, we also tested the protein trap line *Shal^MI00446-GFSTF.1^*, derived from *Shal^MI00446^* by replacing the MiMIC cassette in the insertion with the GFSTF marker cassette flanked by splice acceptor and splice donor sites (Table 1, Extended Data Fig. 2-1) to generate full-length *Shal* proteins with the markers fused in-frame (Nagarkar-Jaiswal et al., 2015). SEPs of this protein trap line were not significantly different from controls (Dunnett's; *p* = 0.60; Table 2) but significantly different from the parent *Shal^MI00446^* allele (Fig. 2*C*; Dunnett's; *p* < 0.0001), suggesting that the fusion protein is fully functional for this phenotype.

Finally, flies expressing the Shal dominant-negative construct in all neurons also showed strong reduction in SEPs compared with controls, (Fig. 2*C*; Dunnett's; *p* < 0.0001; Table 2). Shal dominant-negative SEPs were not significantly different from the stronger insertion alleles, *Shal^MI00446^* and *Shal^f00495^* (Fig. 2*C*; Dunnett's; *p* = 0.97 and *p* = 0.28, respectively) but significantly more reduced than the two weaker insertion alleles, *Shal^MI10881^* and *Shal^MB05249^* (Fig. 2*C*; Dunnett's; *p* = 0.0014 and *p* = 0.022, respectively).

In summary, all mutant or dominant-negative genotypes resulted in significant SEP reductions, and the protein trap fusion restored function to the mutant insertion from which it was derived. Taken together, this indicates that *Shal* plays critical roles in sending auditory signals to the brain.

### Shal is required for tuning antennal active movements

**Figure 3.**
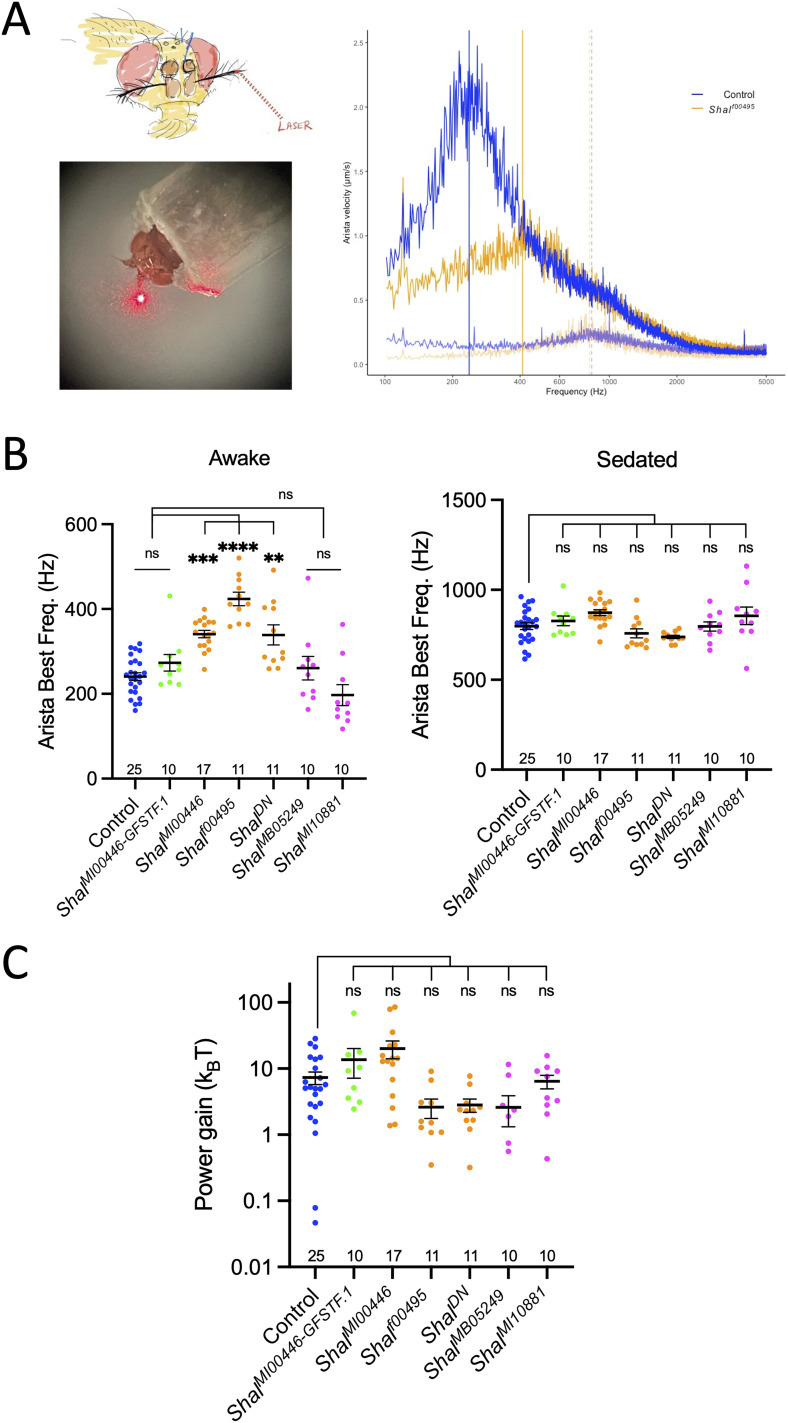
*Shal* loss of function shifts antennal resonant frequency with little effect on power gain. ***A***, LDV preparation. Reflections from a laser beam focused on the arista allow precise recording of antennal movements. In the awake state, the antenna of a control fly (blue trace) shows vibrations in a range of frequencies below 1.5 kHz, with a peak at ∼240 Hz (vertical blue line). The same antenna under CO_2_ sedation (light blue trace) shows lower magnitude vibrations with a peak in the 800 Hz range (light blue dashed vertical line). These recordings in the absence of sound stimuli are called “FF.” Similar laser vibrometry recordings from an awake *Shal^f00495^* mutant (orange trace) shows peak vibrations in the 400 Hz range (orange vertical line), but when sedated, vibrations from the same fly (light orange trace) are in the 800 Hz range (light orange dashed vertical line) resembling a sedated control fly. ***B***, Scatterplots of the peaks (best frequencies) of antennal FF in the awake state (left graph) and the sedated state (right graph). Each dot represents the best frequency of one antenna, and the number of antennae tested for each genotype is indicated at the bottom of each graph. Bars indicate means; error bars represent SEM. Colors of dots match genotypes of Figure 2. In the awake state, the strong alleles (orange dots) show best frequencies significantly higher than controls (blue dots). However, the weak alleles (magenta dots) as well as the *Shal* protein trap (green dots) do not significantly shift the best frequencies compared with controls. Brown–Forsythe ANOVA; *p* < 0.0001; with Dunnett's multiple comparisons (ns, not significant; **p < 0.01; ***p < 0.001; ****p < 0.0001). In the sedated state, none of the genotypes significantly differ from controls. ***C***, A scatterplot of estimated power gain calculations. Genotypes and dot colors as in ***B***. Power gains in *Shal* mutant genotypes do not differ significantly from controls. Kruskal–Wallis ANOVA with Dunn's multiple comparisons (ns, not significant). For plots of *Q* values of LDV data for *Shal* genotypes, see Extended Data Figure 3-1.

To test whether Shal channel activity in the ciliated dendrite is important for active mechanosensation to tune the antenna's resonant frequency as well as energy injection into antennal movement, we measured FF of *Shal* mutant and control fly antennae, both awake and under CO_2_ sedation, using LDV (Göpfert and Robert, 2003; Göpfert et al., 2005; Weinberger et al., 2017; Fig. 3*A*). By fitting a previously published damped harmonic oscillator fit to this LDV data, we were able to estimate key parameters. In awake flies, we found significant differences in resonant frequency based on genotype (Fig. 3*B*; Brown–Forsythe ANOVA; *p* < 0.0001; Table 2). The antennal resonant frequency of awake control flies was 240.9 ± 9.1 Hz (mean ± SEM; Fig. 3*B*). Among *Shal* insertion mutants, *Shal^MB05249^* and *Shal^MI10881^* flies showed no significant change in awake tuning, with 260.6 ± 27.8 and 197.0 ± 24.7 Hz, respectively (Fig. 3*B*; Dunnett's T3 multiple comparisons; *p* > 0.9999 and *p* = 0.84, respectively; Table 2). In contrast, *Shal^f00495^* and *Shal^MI00446^* flies showed significant increases in awake tuning, with 423.7 ± 15.8 and 340.9 ± 8.8 Hz, respectively (Fig. 3*B*; Dunnett's; *p* < 0.0001 each; Table 2). Furthermore, the dominant-negative *Shal* flies also showed increased awake tuning of 338.9 ± 23.6 Hz (Fig. 3*B*; Dunnett's; *p* < 0.033; Table 2). Meanwhile, the *Shal* protein trap flies were not distinguishable from controls, at 273.3 ± 19.4 Hz (Dunnett's; *p* = 0.091; Table 2).

10.1523/ENEURO.0083-24.2024.f3-1 Figure 3-1 **Q factors of LDV data for *Shal* genotypes.** Scatter plots of the Q values, indicating sharpness of the peaks, of antennal free fluctuations from Fig. 3 in the awake state (A) and the sedated state (B). Each dot represents the Q of one antenna recording and the number of antennae tested for each genotype is indicated at the bottom of each graph. Bars indicate means; error bars represent SEM. Colors of dots match genotypes of Fig. 2 and 3. In the awake state, the strong alleles (orange dots) show statistically significantly differences from controls (blue dots). However, the weak alleles (magenta dots) as well as the *Shal* protein trap (green dots) do not significantly shift the Q values compared to controls. Kruskal-Wallis ANOVA, p < 0.0001, p = 0.32 for sedated flies, with Dunn’s multiple comparisons (ns: not significant; *p < 0.05; **p < 0.01; ***p < 0.001). In the sedated state, none of the genotypes significantly differs from controls (Kruskal-Wallis, p = 0.32). Download Figure 3-1, TIF file.

Sedation with CO_2_ removes physiologically active processes, leaving only passive mechanical movements of the antenna (Fig. 3*A*; Göpfert and Robert, 2003; Göpfert et al., 2005; Weinberger et al., 2017). Under sedation, we found the resonant frequency of control flies to be 797.9 ± 18.8 Hz (Fig. 3*B*), as expected from previous reports (Riabinina et al., 2011). We also found all *Shal* genotypes tested to show passive resonant frequencies in the same range (Fig. 3*B*) and not significantly different from controls (Dunnett's multiple-comparison *p* values ranging from *p* = 0.087 to *p* > 0.9999; Table 2), although the overall Brown–Forsythe ANOVA was significant at *p* = 0.0058, primarily attributable to a difference between *Shal^MI00446^* and the Shal dominant-negative flies. These findings show that Shal has minimal impact on passive mechanical properties of the antenna but is required to shift the antennal tuning from the passive 800 Hz range to the fully active 240 Hz range. Without Shal activity in the strongest loss-of-function genotypes, the active tuning shifts only partially, to the 400 Hz range.

Another aspect of tuning is the sharpness of the tuning peak, which can be quantified by estimating the *Q* factor from the damped harmonic oscillator fit. When we calculated *Q* values in the FF recordings from Figure 3*B* in awake flies (Extended Data Fig. 3-1*A*), we found significant differences between control flies and the three genotypes that showed the strongest SEP phenotypes. These differences were not all in the same direction, however, with one line showing an increased tuning sharpness and the others showing decreased tuning sharpness. For sedated flies, we found no differences in *Q* factor between all groups (Extended Data Fig. 3-1*B*; Table 2).

From the active and passive LDV recordings, it is possible to calculate auditory power gain, the active injection of energy into the hearing system representing one measure of the energy provided by the active system above the passive baseline. While a Kruskal–Wallis ANOVA test of power gain calculations (Fig. 3*C*) shows significant differences by genotype (*p* = 0.0015), none of the individual genotypes is significantly different from controls (Fig. 3*C*; Dunn's multiple-comparison *p* values range from *p* = 0.73 to *p* > 0.9999; Table 2). Significance in the overall model arises primarily from *Shal^MI00446^* versus *Shal^f00495^* (Dunn's *p* = 0.02) and *Shal^MI00446^* versus *Shal^MB05249^* (Dunn's *p* = 0.011). Compared with controls, we see no obvious changes in power gain among *Shal* loss-of-function genotypes, suggesting that Shal has little effect on the overall energy that JO neurons generate for active mechanosensation.

## Discussion

We have shown that Shal is expressed in JO neurons where it is important for the neuronal output, as measured by the electrophysiological signals sent to the brain along the antennal nerve. Shal localizes not only to the JO neuron cell bodies but also to the sensory dendrite where it is positioned to participate in active hearing mechanisms. Indeed, our LDV data confirm that loss of Shal significantly affects the active physiological tuning of antennal oscillation in the absence of sound. In wild-type flies, such tuning mechanisms shift antennal oscillations from a resonant frequency in the 800 Hz range to the 240 Hz range.

We were surprised to find that some alleles, specifically *Shal^MI10881^* and *Shal^MB05249^*, reduced SEPs but had little effect on the active mechanisms of antennal movement. This suggests that Shal may have distinct contributions to these active mechanisms, presumably acting in the dendrite, compared with the generation or propagation of the action potential measured in the nerve, functions that may depend more on Shal channels localized in the soma. Thus, these two alleles may differ in the specific properties of the Shal channel required for localization to these two compartments or of the Shal channel K^+^ currents as they contribute to these different functions. Alternatively, these two functions may simply differ in their susceptibility to reduced expression levels. Interestingly, the *Shal^MI10881^* and *Shal^MB05249^* alleles are inserted into the first intron, in the 5′-UTR region, while the other insertion alleles are inserted in the second intron, located in the coding region (Extended Data Fig. 2-1). This difference in the insertion site may allow any read-through transcripts still to generate normal full-length Shal channel proteins for those insertions in the first intron, albeit at reduced levels, while read-through transcripts from those in the second intron will affect the channel structure.

The dominant-negative Shal is associated with a single amino acid change, from tryptophan to phenylamine at Position 362 (Ping et al., 2011), located at the pore region, based on a similar mutation in mouse K_v_4.2 (Barry et al., 1998). K_v_4 channels assemble as tetramers (Kise et al., 2021), and incorporation of K_v_4.2^W362F^ subunits has been shown to block channel activity (Barry et al., 1998). Overexpression of *UAS-HA-Shal^W362F^* in the background of normal *Shal* alleles is likely to result in a preponderance of mutant channel subunits, making assembly of a wild-type tetramer rare and leading to a dominant-negative phenotype equivalent to a strong loss-of-function phenotype (Ping et al., 2011). This interpretation is consistent with our results in both the electrophysiological and antennal movement phenotypes (Figs. 2*C*, 3*B*). We note that our results with the dominant-negative Shal are consistent with our results for insertional loss-of-function alleles for all three phenotypes (localization, electrophysiology, and antenna motion), mitigating concerns of possible off-target effects of the dominant-negative construct. In no case do we make conclusions solely based on the dominant-negative genotype.

The cellular trafficking mechanisms that localize the Shal protein in JO neuron dendrites are unknown. In olfactory projection neurons, dendritic localization of Shal depends on a di-leucine motif and its interacting protein, Shal-interacting Di-leucine protein (SIDL; Diao et al., 2010). The *Shal* locus has been shown to generate three distinct mRNA splicing isoforms which differ in the 3′-end (Butler et al., 1990; Wei et al., 1990; Extended Data Fig. 2-1). The di-leucine is located at residues 481–482, about seven residues before the divergence of the three isoforms. Thus, all three isoforms contain the di-leucine motif, but as the dendritic targeting was tested in the context of the longest isoform, and it is not known which isoforms are expressed in JO neurons, we cannot be certain whether this dendritic targeting mechanism applies to Shal in JO neurons. Furthermore, while *SIDL* is expressed in JO neurons, its expression level is low (Fly Cell Atlas; H. Li et al., 2022). K_v_4.2 and K_v_4.3 localization in the rat hippocampus may rely on multiple mechanisms, including localization of the mRNA based on sequences in the 3′-UTR or protein localization through K_v_ channel-interacting proteins or membrane-spanning dipeptidyl aminopeptidase-like proteins (Sheng et al., 1992; Nadal et al., 2003; Jo et al., 2010; Alfaro-Ruíz et al., 2019). Whether any of these mechanisms play a role in JO neurons remains to be tested.

**Figure 4.**
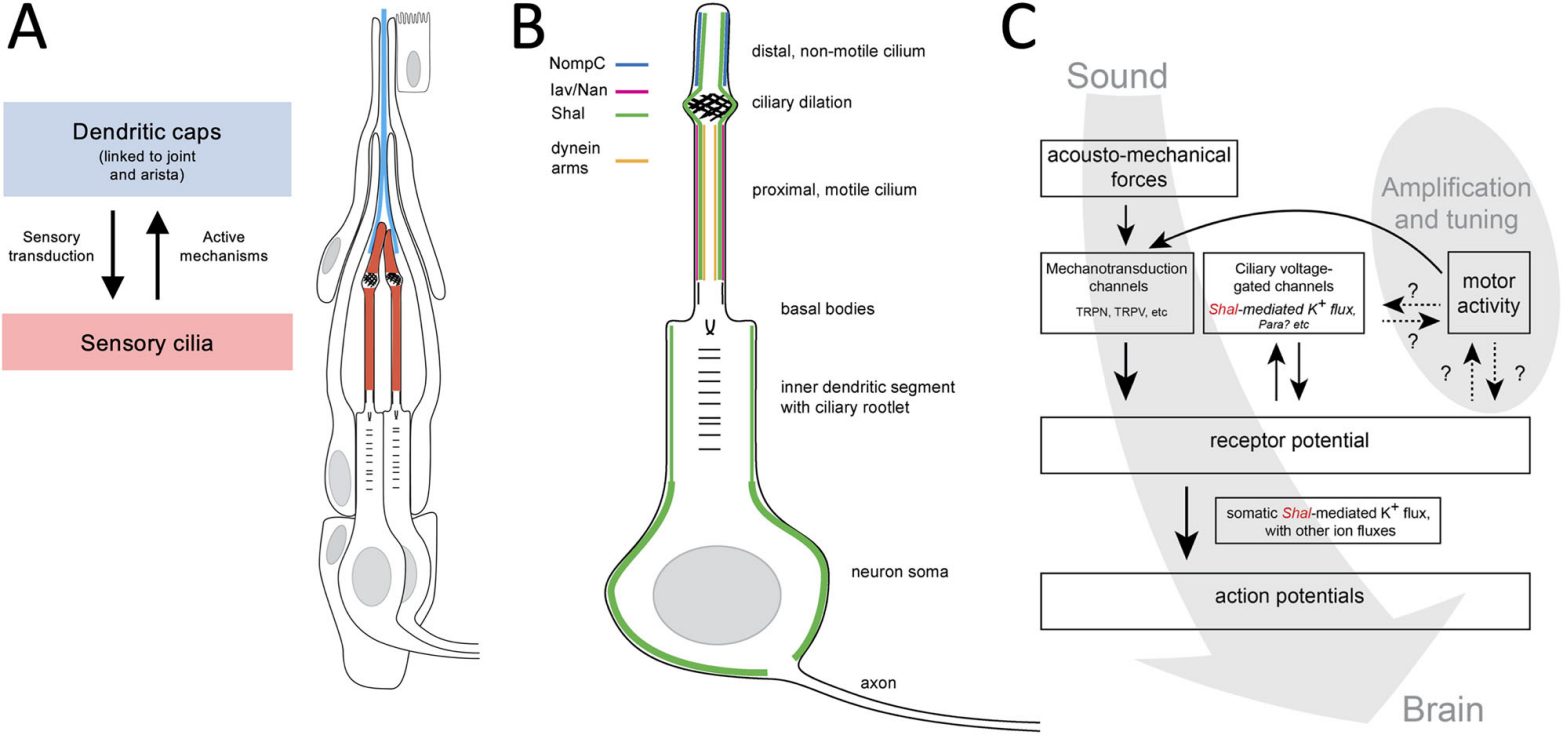
Model of Shal in active mechanotransduction in JO. ***A***, During sensory transduction, sound-induced movements of the arista are transferred by the dendritic caps (blue) to the JO neuron ciliated dendrites (red) to initiate mechanotransduction. In the absence of sound stimuli, active ciliary movements in the sensory dendrites of JO neurons transfer kinetic energy to the antennal joint via the dendritic cap resulting in antennal vibrations (FF). ***B***, In the JO neurons, several ion channels have been localized to the dendritic compartment. NompC (TRPN) is localized in the distal-most ciliary compartment beyond the ciliary dilation. This ciliary segment is nonmotile given the absence of axonemal dynein arms. Nan/Iav (TRPV) channels localize in the motile proximal ciliary segment, colinear with the localization of axonemal dynein arms. In this study, we show that Shal localizes at low intensity along the entire sensory cilium and strongly in the neuron soma membrane. ***C***, Schematic of complex interplay between the dynamics of membrane currents (receptor potentials) mediated by both TRPN and TRPV and the timing of active ciliary movements. Ciliary localization of Shal is consistent with a role in development of receptor potentials and directly or indirectly affecting motor activity. Alternatively, rather than dynamic cycle-by-cycle gating, we cannot rule out the possibility that Shal provides a static bias in membrane potential that impacts TRPN- and TRPV-mediated currents and active ciliary movements. Either way, loss of Shal may change the receptor potential dynamics sufficiently to shift the motor activity timing, shifting the antennal tuning. Altered tuning together with altered receptor potentials may reduce the activation of action potentials at the axon. Localization of Shal in the inner dendritic segment and in the neuron soma may affect the propagation of the sensory receptor potential or its conversion into an action potential. Some alleles may affect this somatic function, disrupting the generation or propagation of full action potentials without affecting active ciliary movements.

The specific mechanism by which dendritic Shal channels shape antennal motion is not known. Active antennal motion is presumably performed by axonemal dyneins (Eberl, 1999; Karak et al., 2015), and their regulation must require appropriate membrane receptor potential dynamics to coordinate the ciliary movement in the correct phase to change the frequency appropriately (Fig. 4). Our findings of minimal effect on power gain in *Shal* mutants (Fig. 3*C*; Table 2), with effects primarily on tuning, suggest the strength of ciliary activity is largely unaffected but is rather out of phase with membrane potential changes, inhibiting tuning to the desired 240 Hz range. Our estimates of *Q* values based on free fluctuation fits (Table 2) found no differences in tuning sharpness across genotypes for sedated flies; however, for awake flies, differences were identified. This could indicate the role of membrane voltage in influencing the mechanobiology of ion channels which determine ear stiffness, such as NompC.

One possible mechanism by which Shal channels might affect receptor potential dynamics locally in the dendrite is to sharpen receptor potentials through their fast activation and inactivation kinetics (Butler et al., 1990; Wei et al., 1990; Baldwin et al., 1991; Jerng et al., 2004; Ping et al., 2011). In hippocampal CA1 pyramidal cell dendrites and granule cell dendrites in the dentate gyrus, the transient A-type potassium currents (encoded by K_v_4) have also been reported to inhibit backpropagation of action potentials, limit the dendritic initiation of action potentials, and dampen the effect of excitatory dendritic inputs (Hoffman et al., 1997; Yang et al., 2015; Oulé et al., 2021). Thus, in JO neuron dendrites, Shal may be shaping the kinetics of one receptor potential as it develops and may also modulate any backpropagation to coordinate with the next cycle of receptor potential activation to sustain active oscillations. Alternatively, we cannot rule out that Shal may bias the membrane currents or receptor potential in a sustained static manner rather than cycle-by-cycle dynamics. Either way, these activities likely also depend on other dendritic ion channels, including NompC and Nan/Iav, as well as potentially the voltage-gated sodium channel encoded by the *para* gene, which has also been shown to localize to JO neuron dendrites (Ravenscroft et al., 2023). The interdependence of Shal-mediated K^+^ fluxes, axonemal dynein-mediated ciliary motor activity, and receptor potentials are depicted in Figure 4*C*. However, there remain many open questions. In particular, it will be important to solidify the specific roles of the TRPV and TRPN channels in the development of the receptor potentials. Furthermore, determining the specific ionic composition of the receptor lymph in the scolopale space is important, which, if K^+^-rich as is commonly thought, may affect the rate and perhaps the direction of K^+^ flux across the ciliary membrane. Regulation of the dynein arm motor activity, especially the mechanisms by which it coordinates with the membrane electrical activity, must also be resolved. Detailed understanding of how all these channel activities interact to shape receptor potentials and modulate the timing of axonemal dynein arm activity to influence antennal motion will require further investigation.

## Synthesis

Reviewing Editor: Luke Remage-Healey, University of Massachusetts Amherst Decisions are customarily a result of the Reviewing Editor and the peer reviewers coming together and discussing their recommendations until a consensus is reached. When revisions are invited, a fact-based synthesis statement explaining their decision and outlining what is needed to prepare a revision will be listed below. The following reviewer(s) agreed to reveal their identity: Jonathan Ashmore, Rupesh Chillale. Note: If this manuscript was transferred from JNeurosci and a decision was made to accept the manuscript without peer review, a brief statement to this effect will instead be what is listed below.

The manuscript was evaluated by two expert referees. While they were generally enthusiastic about the findings and presentation, they raised several important concerns as outlined below.

The reviewers were concerned about the statistical methods and excesses, and the lack of clarity on sample sizes. Also, they were concerned that the authors should obtain a higher resolution for Kv4.2 labeling of dendrites. They are recommending that the authors consider functional outcomes, including behavior, potential interactions of Shal with other ion channel and signaling molecules, off-target effects of a dominant-negative genetic construct (including isoforms), and a biophysical connection between vibrometry data (mechanical) and the K channel data (electrophysiological) in the interpretation/discussion of their work. Lastly, some method details were missing and need to be revisited.

Reviewer 1

This paper describes the evidence for the potassium ion channel Kv4.2 (Shal) in the sensory cells of the Johnston's organ of drosophila - and in particular in the cell body and the sensory dendrites of the cells. In loss of function genotypes, the electrical signal recorded from the JO was reduced by more than three fold (Fig 2) and the paper also reports a reduction of the 'active' tuning of the arista (as shown in Fig 3): this was reduced and the best frequency significantly altered.

This is an interesting case example of how a single ion channel, not necessarily one of those implicated in the specific transduction step, can modify the coding significantly. I was looking for more of model that might explain the relationship between the electrical responses (2B) and the arista behaviour (Fig 3). The discussion here leaves this a little open ended with no clear model. It is clear that the neural resonance amplitude is significantly altered, as well as the quality of the resonance (multiple peaks), but it would be helpful why the second response ~ 15 ms after the pulse train also vanishes.

However some improvement of the evidence in Figure 1 is definitely required. It does not as it stands represent a very compelling case for the localisation presented in summary Figure 5. The Kv4.2 labelling of the dendrites is very weak, possibly even punctate, and hard to distinguish from the background - and there is always the concern that the fluorescent signal is being light piped up the dendrite. A higher resolution image is definitely required. (This is even a case for trying super-resolution optics if available.) The brackets indicating the structures could also certainly be improved - in my copy the blue and orange brackets are barely distinguishable

Further points

1) Figure 2: How was the SEP measured? The min-max difference within one cycle might make sense to reflect the action potentials but cannot meaningfully be applied to the mutant response; so it might be better to use peak negativity of the response instead?

2) Figure 3: It would be helpful to see the raw data for at least one of the mutants - not just the wildtype - to see how the curves change. The Q factor can also be give as well - how did that change with different fly types?

3) Methods: Laser Doppler... mechanical tuning:

l. 361. There appears to be a typo: there should be a free omega in numerator, not under the square root. The peak of this function occurs at w0, so even just fitting the peak of the vibration pattern with a parabola would be sufficient. As it stands, the Q values can also be fitted what were they?

4) Methods: Laser Doppler power gain:

A reference for the definition of l.381 might be helpful. Fig 3A suggests that the vibrometer bandwidth is limited to 1 kHz, in which case the sum of the Fourier coefficients for the passive case is an underestimate, and the power gain is overestimated.

5) Statistics: There a profusion of tests used (e.g. four different tests in ll 197-210 of Results page 2. Are they all necessary? What was being used for the calculation - Prism? As a matter of style, the authors should also consider whether the data justifies 4 significant figures (I personally don't think so). What n values were used for each statistical determination?

Reviewer 2

The paper "The voltage-gated potassium channel Shal (Kv4) contributes to active hearing in Drosophila" presents a study investigating the role of the Shal (Kv4) voltage-gated potassium channel in the auditory mechanotransduction of Drosophila melanogaster. The authors explore the localization of the Shal protein in Johnston's organ (JO) neurons, which are responsible for hearing in Drosophila, and assess its contribution to auditory function and active antennal tuning. They employ a variety of experimental techniques, including GFP-protein trapping, electrophysiological recordings, and laser Droppler vibrometry to investigate the localization and functional role of Shal in JO neurons.

The study provides substantial evidence that Shal is localized in the cell body and the proximal dendrite region of sensory neurons and that it plays a crucial role in coordinating energy-dependent antennal movements essential for tuning the antenna to courtship song frequencies. The findings contribute to a deeper understanding of the ion channels involved in insect auditory mechanotransduction and the mechanisms underlying active hearing in Drosophila.

Major comments:

The study primarily focuses on the cellular and physiological aspects of Shal's role in hearing. However, including behavioral assays to assess the impact of Shal mutations on the flies' ability to respond to sound or participate in courtship behaviors could have provided a more comprehensive understanding of Shal's functional significance.

While the study demonstrates the involvement of Shal in active hearing, it does not delve deeply into the molecular mechanisms by which Shal channels influence the dynamics of auditory transduction currents or how they interact with other ion channels in the sensory dendrites.

The use of genetic manipulations, such as the dominant-negative construct and insertion mutants, might have off-target effects that could influence the observed phenotypes. How did the authors control for off-target effects? Moreover, the authors mentions that the Shal locus generates distinct mRNA splicing isoforms. Did the authors explore the potential differential roles of these isoforms and specific contributions?

I feel that the discussion could have benefited from a comparative analysis of Shal's role in hearing with other known ion channels involved in Drosophila auditory transduction. For example, in the context of [1-3]

Minor:

In Fig-3B the significance of stars could be confusion. I suggest the authors could make changes as depicted in Fig-2C.

In all the figures where statistics are presented, the sample size is not mentioned in any of them.

Methods could be improved by adding a statistical analysis section and detail description of analysis conducted.

1. On TRPN1 (NompC):

Effertz, T., Wiek, R., &Göpfert, M. C. (2011). NompC TRP channel is essential for Drosophila sound receptor function. Current Biology, 21(7), 592-597.

2. On TRPV (Inactive and Nanchung):

Gong, Z., et al. (2004). Two interdependent TRPV channel subunits, Inactive and Nanchung, mediate hearing in Drosophila. The Journal of Neuroscience, 24(41), 9059-9066.

3. On Piezo:

Kim, S. E., Coste, B., Chadha, A., Cook, B., &Patapoutian, A. (2012). The role of Drosophila Piezo in mechanical nociception. Nature, 483(7388), 209-212.

## Author Response

Response to Reviews Dear Editor:

We are grateful to you and to the anonymous reviewers for positive comments and constructive criticism on our manuscript "The voltage-gated potassium channel Shal (Kv4) contributes to active hearing in Drosophila", by Gregory et al. As a result, we have made substantial changes to the manuscript and the figures, and we believe the manuscript is now much improved.

Detailed responses to the synthesis statement and to the specific reviewers' comments are listed below.

Synthesis statement:

The manuscript was evaluated by two expert referees. While they were generally enthusiastic about the findings and presentation, they raised several important concerns as outlined below.

The reviewers were concerned about the statistical methods and excesses, and the lack of clarity on sample sizes.

We have added a dedicated section in the Materials and Methods detailing our statistical analysis (Lines 174-188).

We also applied estimation statistics to each of our graphed data panels, and we included them as Table 2. We retained the ANOVA statistics because they provide very similar outcomes and conclusions, but with more granularity. For example, in Fig. 2C, the estimationstats.com website only allows comparison of all genotypes to a shared control, but not comparisons between alleles. We believe therefore that the statistical approach we used is useful.

We have included sample sizes for each genotype on each graph, as well as confirmed their appropriate inclusion in the text.

Also, they were concerned that the authors should obtain a higher resolution for Kv4.2 labeling of dendrites.

We agree with the reviewers that the stainings in Figure 1 could be improved.

We acquired 3 additional antibodies, including the two mouse Kv4.2 antibodies from DSHB, but we were not able to obtain convincing staining even in the soma membrane, consistent with insufficient conservation in the epitope sequences.

Thus, we sought to improve the stainings with the protein trap allele using antibodies against both EGFP and FLAG tags (shown in the new Fig. 1A), and with the HA-tagged dominant-negative construct using anti-HA antibodies (shown in the new Fig. 1B). Both staining approaches gave similar results, with strong staining in the soma region, and weaker but, in our opinion, convincing staining in the sensory cilium.

They are recommending that the authors consider functional outcomes, including behavior, potential interactions of Shal with other ion channel and signaling molecules, off-target effects of a dominant-negative genetic construct (including isoforms), and a biophysical connection between vibrometry data (mechanical) and the K channel data (electrophysiological) in the interpretation/discussion of their work.

We thank the reviewers for all these considerations. Regarding behavioral experiments, we agree that there can be a variety of downstream outcomes of loss of Shal function, but we expect that as these outcomes will be far downstream, they will not help in addressing the active mechanosensation mechanisms under investigation. Shal is expressed in many central neurons, so effects on the behaviors will be difficult to ascribe specifically to defects in JO.

We are also interested in potential interactions between Shal and other ion channels, especially the mechanosensitive ion channels TRPN and TRPV, but our data do not allow any such conclusions to be made without adding a whole new dimension of experiments, which are beyond the scope of the current manuscript.

We also share the reviewers' concerns regarding off-target effects of the DN, specifically because this construct is overexpressed. Our concerns are mitigated by the fact that our results with the DN construct confirm our other results with loss of function alleles (for localization, electrophysiology and LDV phenotypes), and we do not make any conclusions from the data for this genotype alone. We have added sentences to the Discussion to reflect this (Lines 331-335).

Finally, we share the reviewers' desire to understand the biophysical connection between the mechanical and electrophysiological data. To this end we added a schematic in Figure 4C to help illustrate the interconnectivity between these factors. There remain many biophysical questions that we hope further studies will inform.

Lastly, some method details were missing and need to be revisited.

See details in items below.

Reviewer 1 This paper describes the evidence for the potassium ion channel Kv4.2 (Shal) in the sensory cells of the Johnston's organ of drosophila - and in particular in the cell body and the sensory dendrites of the cells. In loss of function genotypes, the electrical signal recorded from the JO was reduced by more than three fold (Fig 2) and the paper also reports a reduction of the 'active' tuning of the arista (as shown in Fig 3): this was reduced and the best frequency significantly altered.

This is an interesting case example of how a single ion channel, not necessarily one of those implicated in the specific transduction step, can modify the coding significantly.

Thank you for the positive comments! I was looking for more of model that might explain the relationship between the electrical responses (2B) and the arista behaviour (Fig 3). The discussion here leaves this a little open ended with no clear model.

As mentioned above, we have now included a schematic in Fig. 4 to help illustrate the intimate interconnectivity between Shal-mediated K+ fluxes, receptor potentials mediated by all combined ion channel and transporter activity and the dynein-mediated ciliary activity underlying the active mechanics. We could speculate about biophysical mechanisms, but we feel that there are still so many key unknowns that make this a house-of-cards exercise that would lead to more distraction than useful mechanisms.

For example, would K+ flux be inward or outward, given the putative high K+ in the receptor lymph, which unfortunately is very difficult to measure in chordotonal organs? The contributions of TRPN and TRPV to the initiation and dynamic development of the receptor potentials are still unclear despite 2 decades of work. The motor mechanisms themselves, which point almost certainly to axonemal dynein motors, are also still opaque, especially even the most basic understanding of how these multi-protein complexes are regulated, let alone how these regulatory mechanisms could synchronize to the electrical activity.

We believe that our data make an important contribution by identifying an ion channel previously unsuspected to contribute to both the mechanical and electrical sensory activity and will need to be accounted for in any future such models. However, we feel that it is too early to speculate on these mechanisms in more detail without further data to support them. We have expanded the text (Lines 375-383) to reflect these ideas.

It is clear that the neural resonance amplitude is significantly altered, as well as the quality of the resonance (multiple peaks), but it would be helpful why the second response ~ 15 ms after the pulse train also vanishes.

We have now included an additional trace in Fig. 2B to help illustrate the mutant phenotype, but also the variation in the exact shape of the response pulses, which can vary slightly due to the exact electrode placement in these extracellular field recordings. This shape variation averages out over the recordings from 10 or so antennae per genotype, but the amplitude as we measure it has proven a reliable measure of auditory response.

The major response pulses, which begin about 7 ms after the stimulus, typically have about 4 peaks, consistent with approximately 2 cycles of oscillation in the stimulus. The 7 ms delay is almost entirely acoustic (sound traveling from the loudspeaker to the preparation through the tubing). The minor responses at 15 ms are acoustic echo artefacts (some sound energy reflects from the preparation, travels back to the speaker and reflects back to the preparation), and in fact there are secondary echoes at about 30 ms. Several factors contribute to the relative strength of the echoes, including slight differences in the shape and positioning of the preparation. We added a note in the figure legend to indicate the echo artefacts.

The more reduced example trace (orange) from Shalf00495 still shows the echoes more than the less reduced example trace (magenta) from ShalMI10881. Because we are familiar with these variations, we are cautious not to over-interpret small variations in individual traces.

However some improvement of the evidence in Figure 1 is definitely required. It does not as it stands represent a very compelling case for the localisation presented in summary Figure 5. The Kv4.2 labelling of the dendrites is very weak, possibly even punctate, and hard to distinguish from the background - and there is always the concern that the fluorescent signal is being light piped up the dendrite. A higher resolution image is definitely required. (This is even a case for trying super-resolution optics if available.) The brackets indicating the structures could also certainly be improved - in my copy the blue and orange brackets are barely distinguishable.

We thank the reviewer for these comments regarding the localization data. We have now provided new images upon further staining experiments.

As mentioned above, we tried every available antibody without success, but using the same antibodies as before, we obtained images that we believe more convincingly show ciliary localization. We agree that the labeling is weak in comparison to that in the soma, but we are convinced by the low signal in the cilium. We can rule out that the signal is from a light-piping effect because other antibodies such as monoclonal 22C10 label the inner dendritic segment quite strongly, with no signal in the cilium whatsoever. If light-piping occurs in this preparation, we would see a low level signal in the cilium with 22C10. Many publications that use 22C10 show this, and we have also done 22C10 stains to confirm the same in our hands and with our equipment.

We have improved the brackets labeling the cilium and the inner dendritic segment.

Further points 1) Figure 2: How was the SEP measured? The min-max difference within one cycle might make sense to reflect the action potentials but cannot meaningfully be applied to the mutant response; so it might be better to use peak negativity of the response instead? As explained above, the shape of the pulse responses can vary with slight differences in electrode positioning, such that the ratio of positive peak height to negative peak depth is not a measure that we feel is sufficiently reliable to use alone.

For this reason, we include the height of both the negative and positive components to score the response amplitude. While we would love to have intracellular recordings for a more detailed understanding of the electrical response, we here rely on the field recordings as a JO neuron population-wide response, and we test a sufficient number of animals to capture the biological variation, given the technical variation.

Using the overall max-min difference is reliable because, as shown in Fig. 2, it avoids over-interpreting the data.

2) Figure 3: It would be helpful to see the raw data for at least one of the mutants - not just the wildtype - to see how the curves change. The Q factor can also be give as well - how did that change with different fly types? Thank you for these suggestions. We have included in Fig. 3A both control and Shal mutant LDV profiles to illustrate the differences.

In addition, we captured LDV data up to 10 kHz, so we extended the x-axis up to 5 kHz to better display the data, to balance between depicting sufficient range and sufficient resolution.

Finally, our standard analysis of fitting damped harmonic oscillator curves to each LDV recording provides the computed Q factors, which we have now presented in Extended Data Fig. 3-1.

3) Methods: Laser Doppler... mechanical tuning: l. 361. There appears to be a typo: there should be a free omega in numerator, not under the square root. The peak of this function occurs at w0, so even just fitting the peak of the vibration pattern with a parabola would be sufficient. As it stands, the Q values can also be fitted what were they? For the equation, we used a variant of the equation published by Gopfert et al 2005 PNAS, derived for X ˙ rather than X^2. Indeed, as the reviewer highlighted, the omega should be in the numerator and not the denominator - this reflects an error in the manuscript presentation rather than in our fitting equation, which we have confirmed is correct. We have now fixed the error in the manuscript to align with the actual equation used for fitting.

See previous response regarding Q factors - these values are now included in Extended Data Fig. 3-1.

4) Methods: Laser Doppler power gain:

A reference for the definition of l.381 might be helpful. Fig 3A suggests that the vibrometer bandwidth is limited to 1 kHz, in which case the sum of the Fourier coefficients for the passive case is an underestimate, and the power gain is overestimated.

All LDV recordings were made in the range 1Hz to 10 kHz, with the damped harmonic oscillator curve-fitting done over the range 100Hz to 10kHz to exclude low frequency noise. The fluctuation power was then calculated for these fit curves over the range 1Hz to 10kHz, matching the recording bandwidth. As such we do not believe we have underestimated the fluctuation power for the passive recordings, as by 10kHz there is little difference between the active and passive aristal displacements.

Whilst differences in the active tuning of fly ears were observed in the active state, in the sedated state we did not identify differences between control and mutant ears. If overestimation occurred therefore, it is unlikely to influence comparisons between genotypes.

Previous estimates of power gain have varied between papers and lines (4.6 kBT in Gopfert et al 2005, 7 kBT in Weinberger et al 2017, 10 kBT in Keder et al 2020). Our estimates here for controls of approximately 8kBT align closely with these previous calculations.

We have depicted more of the vibrometer response curves (up to 5 kHz) in Fig. 3A.

5) Statistics: There a profusion of tests used (e.g. four different tests in ll 197-210 of Results page 2. Are they all necessary? What was being used for the calculation - Prism? As a matter of style, the authors should also consider whether the data justifies 4 significant figures (I personally don't think so). What n values were used for each statistical determination? We have now included a Statistical Analysis section in the Materials and Methods which explains our use of those tests. There are two ANOVA tests, depending on whether they rely on parametric or non-parametric approaches. Each of these ANOVA tests has its own multiple comparisons test.

As mentioned in the synthesis section above, we also calculated estimation statistics as recommended by the journal and included them as Table 2.

We agree that we used excessive significant digits in some cases and have modified those accordingly. We have also included all n values on each graph and in Table 2.

Reviewer 2 The paper "The voltage-gated potassium channel Shal (Kv4) contributes to active hearing in Drosophila" presents a study investigating the role of the Shal (Kv4) voltage-gated potassium channel in the auditory mechanotransduction of Drosophila melanogaster. The authors explore the localization of the Shal protein in Johnston's organ (JO) neurons, which are responsible for hearing in Drosophila, and assess its contribution to auditory function and active antennal tuning. They employ a variety of experimental techniques, including GFP-protein trapping, electrophysiological recordings, and laser Droppler vibrometry to investigate the localization and functional role of Shal in JO neurons.

The study provides substantial evidence that Shal is localized in the cell body and the proximal dendrite region of sensory neurons and that it plays a crucial role in coordinating energy-dependent antennal movements essential for tuning the antenna to courtship song frequencies. The findings contribute to a deeper understanding of the ion channels involved in insect auditory mechanotransduction and the mechanisms underlying active hearing in Drosophila.

We are grateful to the reviewer for these positive comments.

Major comments:

The study primarily focuses on the cellular and physiological aspects of Shal's role in hearing. However, including behavioral assays to assess the impact of Shal mutations on the flies' ability to respond to sound or participate in courtship behaviors could have provided a more comprehensive understanding of Shal's functional significance.

Please see our response in the Synthesis Statement section above.

While the study demonstrates the involvement of Shal in active hearing, it does not delve deeply into the molecular mechanisms by which Shal channels influence the dynamics of auditory transduction currents or how they interact with other ion channels in the sensory dendrites.

Please see our response in the Synthesis Statement section above.

The use of genetic manipulations, such as the dominant-negative construct and insertion mutants, might have off-target effects that could influence the observed phenotypes. How did the authors control for off-target effects? Moreover, the authors mentions that the Shal locus generates distinct mRNA splicing isoforms. Did the authors explore the potential differential roles of these isoforms and specific contributions? Please see our response in the Synthesis Statement section above.

I feel that the discussion could have benefited from a comparative analysis of Shal's role in hearing with other known ion channels involved in Drosophila auditory transduction. For example, in the context of [1-3].

Please see our response in the Synthesis Statement section above. We have added a schematic in Fig 4 and enhanced the discussion (Lines 375 -383).

Minor:

In Fig-3B the significance of stars could be confusion. I suggest the authors could make changes as depicted in Fig-2C.

We have applied the same approach using asterisks to denote levels of significance in Fig. 2 and 3.

In all the figures where statistics are presented, the sample size is not mentioned in any of them.

We have added n values to all data in graphs and Table 2.

Methods could be improved by adding a statistical analysis section and detail description of analysis conducted.

We have added a Statistical Analysis section to the Methods section.

1. On TRPN1 (NompC):

Effertz, T., Wiek, R., &Göpfert, M. C. (2011). NompC TRP channel is essential for Drosophila sound receptor function. Current Biology, 21(7), 592-597.

2. On TRPV (Inactive and Nanchung):

Gong, Z., et al. (2004). Two interdependent TRPV channel subunits, Inactive and Nanchung, mediate hearing in Drosophila. The Journal of Neuroscience, 24(41), 9059-9066.

3. On Piezo:

Kim, S. E., Coste, B., Chadha, A., Cook, B., &Patapoutian, A. (2012). The role of Drosophila Piezo in mechanical nociception. Nature, 483(7388), 209-212.
