# Supplementary figures and images for: The Voltage-Gated Potassium Channel Shal (Kv4) Contributes to Active Hearing in Drosophila
Source: eNeuro. 2025 Jan 3;12(1):ENEURO.0083-24.2024. doi: 10.1523/ENEURO.0083-24.2024 (PMC11728854; doi:10.1523/ENEURO.0083-24.2024)

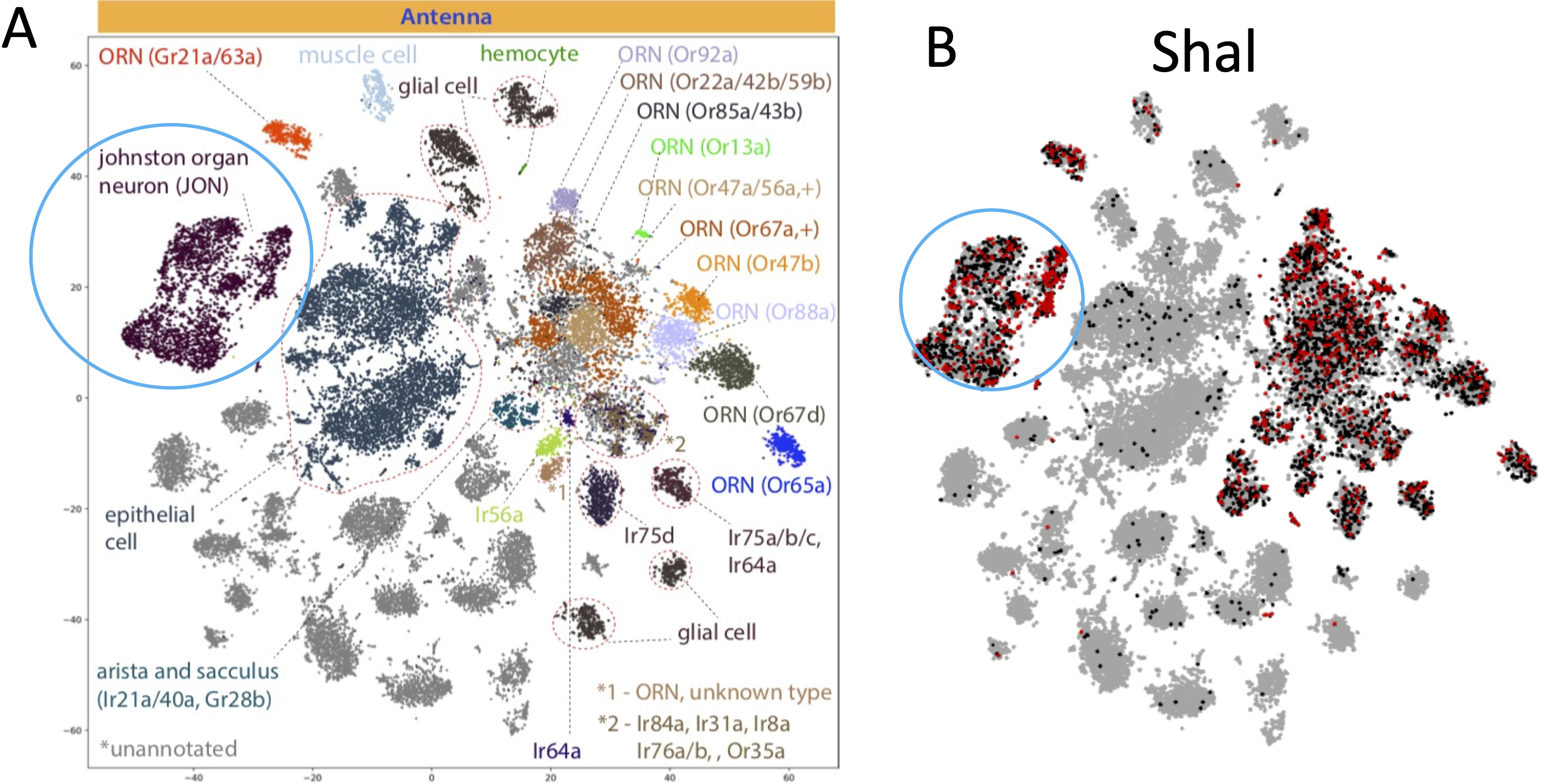

Supplement: Figure 1-1 — Expression of Shal in antennal single nucleus RNA sequencing (Fly Cell Atlas) A. Annotated clustering of single-nucleus RNA transcript expression from antenna (reproduced from Li et al. (2022) with permission), showing a cluster of cells representing the JO neurons (circled). B. Expression of Shal (red) depicted over the same clusters indicates that Shal is expressed in JO neurons (circled) as well as olfactory neurons, using SCope (https://scope.aertslab.org/) (Davie et al., 2018). Download Figure 1-1, TIF file. [file eneuro-12-ENEURO.0083-24.2024-s002.tif]

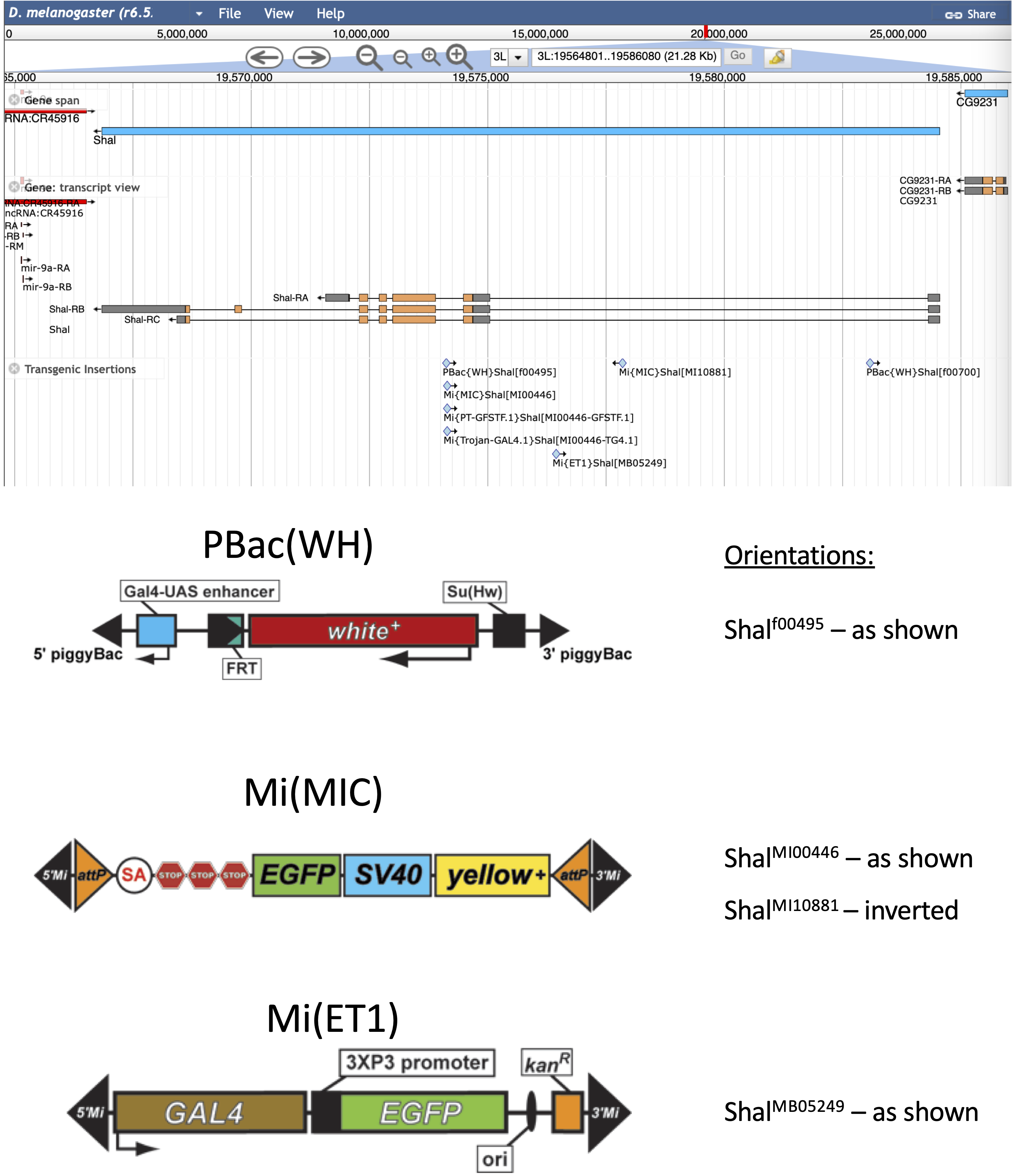

Supplement: Figure 2-1 — Map of Shal locus. Upper panel shows a screenshot of the JBrowse genome browser depicting the Shal locus on chromosome 3L. Shal is transcribed in the leftward direction, with three transcript splice isoforms (coding regions in orange boxes, non-coding regions in gray). Transposon insertion sites are depicted by small blue triangles, labeled. Corresponding transposon structures are diagrammed below (from the Gene Disruption Project (https://flypush.research.bcm.edu/pscreen/transposons.html)), with orientation information relative to the map. Download Figure 2-1, TIF file. [file eneuro-12-ENEURO.0083-24.2024-s003.tif]

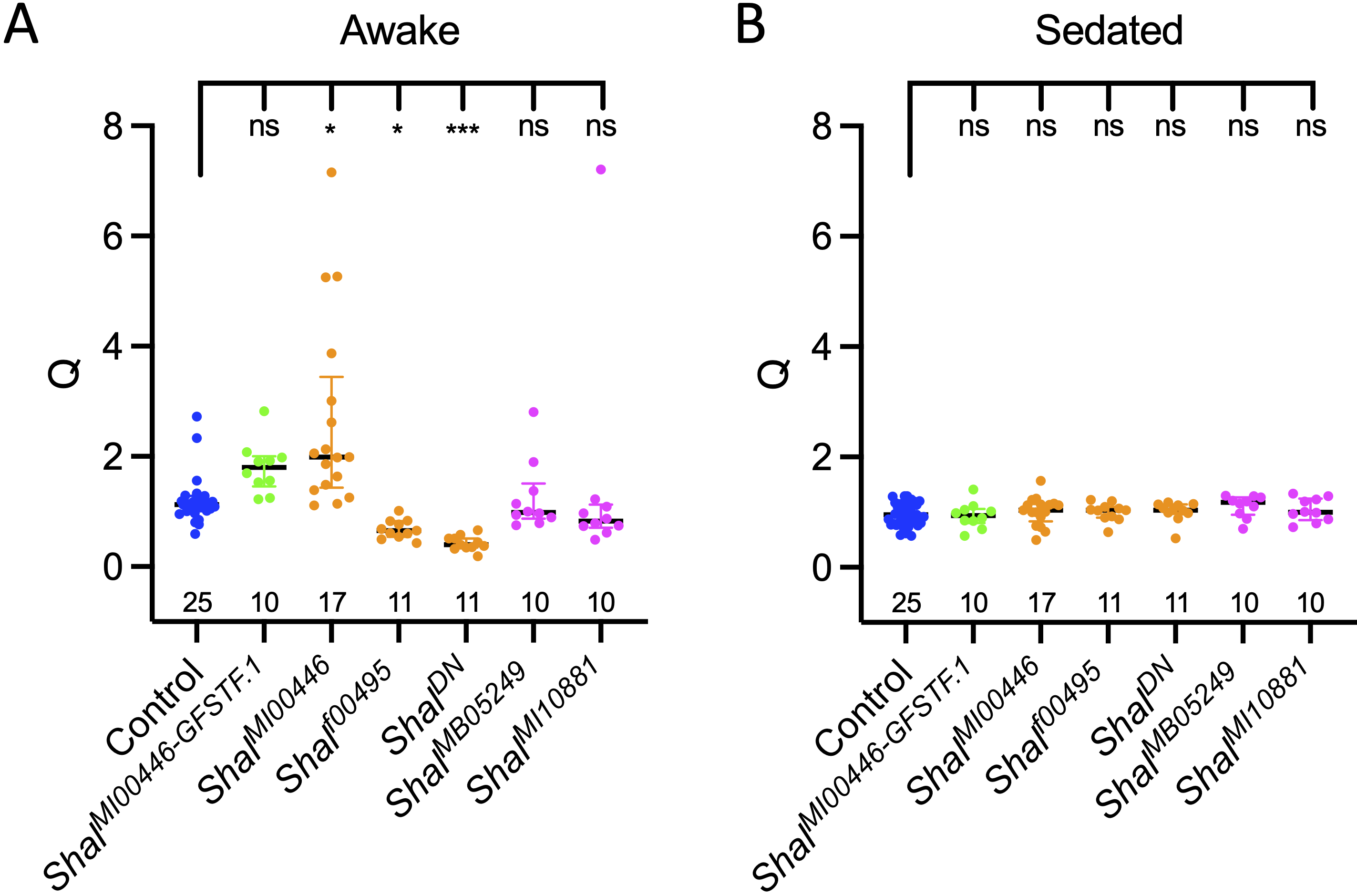

Supplement: Figure 3-1 — Q factors of LDV data for Shal genotypes. Scatter plots of the Q values, indicating sharpness of the peaks, of antennal free fluctuations from Fig. 3 in the awake state (A) and the sedated state (B). Each dot represents the Q of one antenna recording and the number of antennae tested for each genotype is indicated at the bottom of each graph. Bars indicate means; error bars represent SEM. Colors of dots match genotypes of Fig. 2 and 3. In the awake state, the strong alleles (orange dots) show statistically significantly differences from controls (blue dots). However, the weak alleles (magenta dots) as well as the Shal protein trap (green dots) do not significantly shift the Q values compared to controls. Kruskal-Wallis ANOVA, p < 0.0001, p = 0.32 for sedated flies, with Dunn’s multiple comparisons (ns: not significant; *p < 0.05; **p < 0.01; ***p < 0.001). In the sedated state, none of the genotypes significantly differs from controls (Kruskal-Wallis, p = 0.32). Download Figure 3-1, TIF file. [file eneuro-12-ENEURO.0083-24.2024-s004.tif]
